# Supplementary material for: Eupatilin Improves Cilia Defects in Human CEP290 Ciliopathy Models
Source: Cells. 2023 Jun 7;12(12):1575. doi: 10.3390/cells12121575 (PMC10297203; doi:10.3390/cells12121575)
Supplement: Supplementary file 1 [file cells-12-01575-s001.zip › cells-2360732-supplementary.pdf]

Supplemental material

Eupatilin improves cilia defects in human CEP290 ciliopathy models

Corral-Serrano, Sladen, Ottaviani et al.

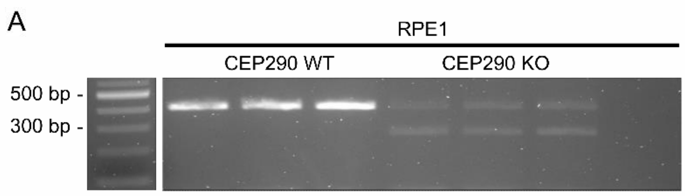

Supplementary Figure S1. Exon 6 skipping in the CEP290 KO RPE1 cell line. A, To detect skipping of *CEP290* exon 6, primers were designed to CEP290 with forward primer in exon 3 and reverse primer in exon 9. RT-PCR was performed on CEP290 WT and CEP290 KO RPE1 cells cDNA. Two different transcripts were observed in the CEP290 KO cell line, indicating exon 6 skipping. CEP290 exon 6 is 144 bp long, which corresponds to the size of the lower band compared to the upper band.

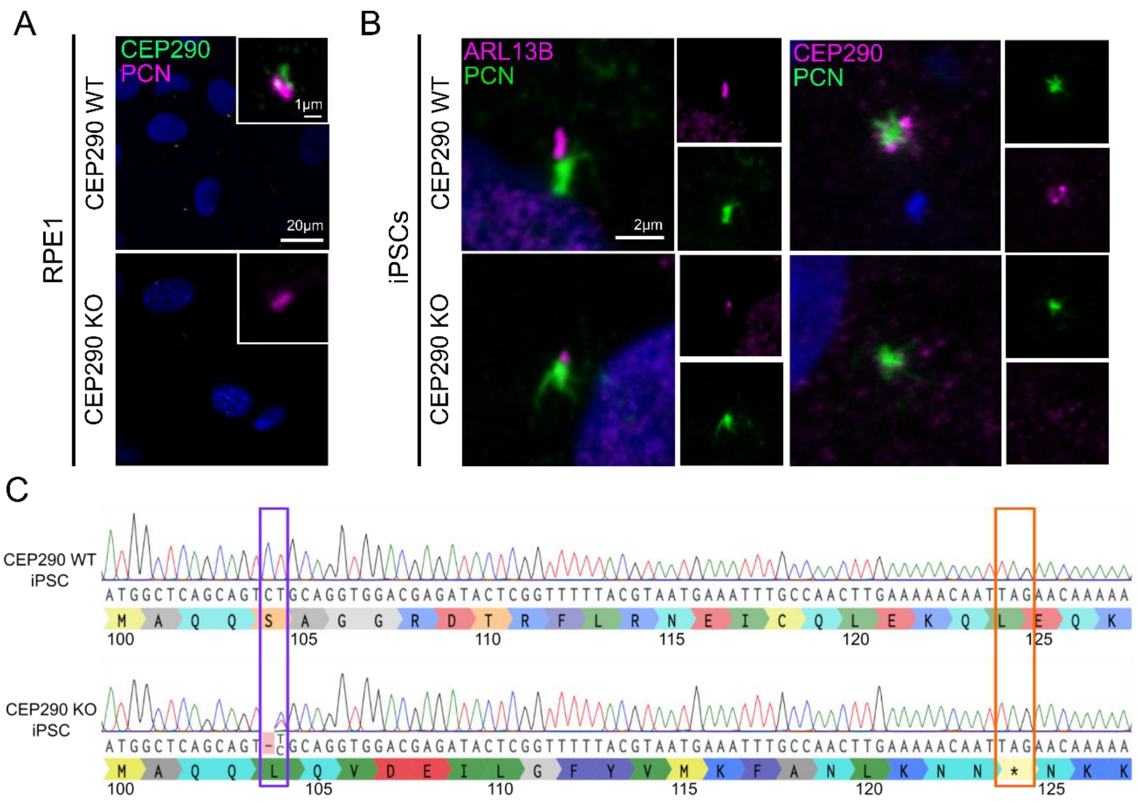

Supplementary Figure S2. CEP290 localization in CEP290 RPE1 and iPSCs KO cells. A, Immunofluorescence staining of CEP290 WT and CEP290 KO RPE1 cells showing cilia staining of pericentrin (PCN, magenta) and CEP290 (green). CEP290 staining shows

expression in the CEP290 WT line while the CEP290 protein is absent in the cilia of CEP290 KO RPE1. Insets show a magnification of both channels. Scale = 1 µm. **B**, Immunofluorescence staining of CEP290 WT and CEP290 KO iPSC showing cilia staining of: upper panels, ARL13B (magenta) and pericentrin (PCN, green); lower panels CEP290 (magenta) and pericentrin (PCN, green). CEP290 staining shows expression in the CEP290 WT while the CEP290 protein is absent in the cilia of CEP290 KO iPSC. Insets show the split of both channels. Scale = 2 µm. **C,** Production of CEP290 KO iPSC. Sanger sequence trace showing compound heterozygous 1 bp deletion (purple box) in CEP290 KO iPSC leading to frame shift and premature stop codon (orange box).

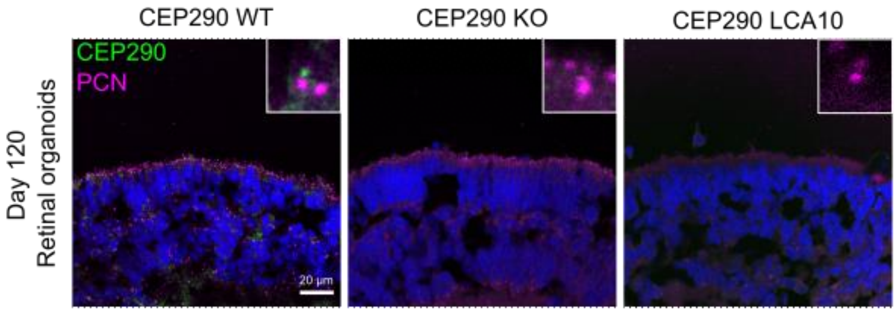

Supplementary Figure S3. CEP290 protein localization in retinal organoids. CEP290 antibody staining in day 120 retinal organoids shows absence of CEP290 in both CEP290 KO and CEP290 LCA10 organoids. Organoids were stained with a CEP290 antibody (green) and a PCN antibody (magenta). Scale bar = 20 μm.

Supplementary Table S1. List of primers used in this study.

qPCR

Gene	Primer	
	Forward	Reverse
ACTIN	CCAACCGCGAGAAGATGA	CCAGAGGCGTACAGGGATAG
ARR3	CAGCTCAGCCCCTACAACTC	ACTGCAAAGCTCTGGGAGAA
CRX	TTTGCCAAGACCCAGTACC	GTTCTTGAACCAAACCTGAACC
GAPDH	CCCCACCACACTGAATCTCC	GGTACTTTATTGATGGTACATGACAAG
OPN1LW/ OPN1MW	CATCCGAGCGGTGGCAAAGC	CAGCAGAATGCCAGGACCATC
RECOVERIN	AGCTCCTTCCAGACGATGAA	CAAACTGGATCAGTCGCAGA
RHO	GGTGGTGTGTAAGCCCATGA	CCTCGGGGATGTACCTGGAC

CEP290 sequencing

Gene	Primer	
	Forward	Reverse
CEP290 exon 6 pair 1	GGCCCAGTGGTTACAACAG	GGTGATGACAAAATGAACAGTGA
CEP290 exon 6 pair 2	CTTTGCATCTCTGGGTGGCTC	ACCAATCTTCCAGACAAAAGCA

CEP290 exon 6 skipping

Gene	Primer	
	Forward	Reverse
CEP290 exons 3 - 9	CAAGAAGTGGAGCTGGCTTTG	CACTGTCTTCCCCTCTTCTTG
